# Supplementary material for: Assessment of Oxidative Stress Markers in Hypertensive Patients under the Use of Renin-Angiotensin-Aldosterone Blockers
Source: Antioxidants (Basel). 2023 Mar 25;12(4):802. doi: 10.3390/antiox12040802 (PMC10135118; doi:10.3390/antiox12040802)
Supplement: Supplementary file 1 [file antioxidants-12-00802-s001.zip › antioxidants-2216579-supplementary.pdf]

Table S1. Results of 24-h ambulatory blood pressure monitoring at every hour.

(a) All hypertensive patients.

Percentile												Time (hours)												
SBP	*23h*	*00h*	*01h*	*02h*	*03h*	*04h*	*05h*	*06h*	*07h*	*08h*	*09h*	*10h*	*11h*	*12h*	*13h*	*14h*	*15h*	*16h*	*17h*	*18h*	*19h*	*20h*	*21h*	*22h*
p25	110.0	107.5	101.4	101.3	102.8	99.9	104.0	104.0	111.3	116.7	122.0	123.0	120.5	122.0	122.6	122.5	119.1	119.5	123.3	121.4	124.6	122.0	120.2	117.8
p50	117.5	115.0	112.0	111.5	112.0	113.3	114.5	114.5	121.5	129.5	134.0	132.0	130.0	129.0	130.4	128.7	128.0	129.3	131.0	129.7	133.0	129.7	129.7	125.0
p75	127.0	124.5	119.0	120.8	121.3	121.3	123.5	125.0	133.3	139.0	141.7	142.8	142.5	141.2	140.3	139.3	140.8	136.9	142.3	138.3	139.8	139.0	139.0	138.2
DBP																								
p25	62.5	61.4	56.5	59.0	58.8	59.0	59.8	60.1	66.3	71.3	0.0	74.0	74.5	76.6	76.7	75.5	71.8	71.5	72.9	73.2	73.0	73.9	72.1	69.7
p50	69.0	66.8	64.3	64.5	65.0	66.0	67.0	67.0	72.8	81.0	75.3	83.0	82.0	84.0	82.6	81.0	80.5	81.0	81.0	81.0	82.0	81.8	81.0	77.5
p75	78.0	73.6	70.3	70.0	72.3	73.5	73.0	76.8	82.9	88.3	85.3	89.5	88.7	90.4	88.8	88.0	86.4	86.9	89.0	89.0	89.0	87.8	89.0	86.8

(b) Patients with morning time use of RAAS blockers.

Percentile												Time (hours)												
SBP	*23h*	*00h*	*01h*	*02h*	*03h*	*04h*	*05h*	*06h*	*07h*	*08h*	*09h*	*10h*	*11h*	*12h*	*13h*	*14h*	*15h*	*16h*	*17h*	*18h*	*19h*	*20h*	*21h*	*22h*
p25	110.5	106.6	98.4	98.8	98.8	98.4	99.3	100.0	109.1	114.8	123.0	123.0	122.7	123.5	123.8	123.0	119.3	119.0	121.8	120.2	124.9	121.3	122.6	119.4
p50	120.5	114.0	109.8	111.5	110.8	113.3	113.0	113.0	120.3	127.7	134.0	130.8	130.0	131.7	128.3	126.8	129.0	129.7	129.7	129.0	133.8	125.7	129.7	125.0
p75	130.4	121.1	117.6	120.8	117.3	119.3	121.5	125.5	130.4	135.3	141.5	141.0	139.0	140.5	138.6	136.0	139.7	137.5	140.1	137.8	140.6	139.0	138.8	140.5
DBP																								
p25	65.1	61.9	56.5	57.5	58.5	59.3	59.8	59.8	66.4	74.8	0.0	77.0	80.0	81.9	78.4	78.3	73.8	73.5	74.2	75.7	77.1	77.3	73.2	71.3
p50	73.8	66.0	64.0	62.8	64.3	66.3	65.5	67.0	77.0	82.5	73.0	85.0	86.0	86.0	84.3	81.4	80.8	83.9	80.9	81.0	85.2	83.0	85.0	81.0
p75	83.3	73.6	70.3	69.6	70.5	77.6	75.5	81.8	84.8	88.3	85.3	91.3	89.0	92.5	88.7	89.1	86.8	89.4	89.3	89.7	90.9	89.3	90.0	89.5

(c) Patients with bedtime time use of RAAS blockers.

Percentile												Time (hours)												
SBP	*23h*	*00h*	*01h*	*02h*	*03h*	*04h*	*05h*	*06h*	*07h*	*08h*	*09h*	*10h*	*11h*	*12h*	*13h*	*14h*	*15h*	*16h*	*17h*	*18h*	*19h*	*20h*	*21h*	*22h*
p25	110.0	108.1	108.3	102.5	104.0	103.1	107.8	110.0	112.1	118.0	122.0	122.0	120.1	119.3	121.2	122.0	118.7	118.6	124.0	121.3	122.0	123.0	118.4	116.3
p50	114.0	117.0	112.5	112.0	113.5	113.5	118.8	119.0	121.5	133.0	133.5	132.5	130.3	128.5	131.8	129.3	127.0	128.3	133.0	130.0	132.0	132.0	129.0	124.7
p75	123.0	129.4	119.0	121.0	123.5	122.0	125.6	125.0	135.5	144.8	145.5	146.1	145.0	142.3	145.3	142.7	145.3	137.3	144.5	141.3	139.3	146.0	140.5	137.0
DBP																								
p25	59.0	60.3	56.8	60.0	59.0	59.0	60.1	60.5	66.3	68.7	0.0	73.6	70.5	74.3	74.4	68.7	67.7	68.3	68.7	68.0	69.3	72.7	71.1	69.7
p50	66.0	67.0	65.3	67.0	66.0	65.8	68.0	67.0	72.2	80.1	77.0	81.7	80.5	81.0	81.3	80.0	79.7	80.2	82.0	80.0	80.0	81.0	77.2	74.0
p75	73.0	74.1	70.8	70.0	72.5	72.8	72.8	73.0	76.5	88.5	85.5	87.5	86.3	89.0	88.8	87.0	86.5	84.1	87.3	87.0	87.0	86.0	86.8	84.0
